# Editorial: Fungal Genetics in Plant Biomass Conversion

**DOI:** 10.3389/fmicb.2022.875768

**Published:** 2022-03-25

**Authors:** Jiwei Zhang, Guodong Liu, Yu Fukasawa

**Affiliations:** ^1^Department of Bioproducts and Biosystems Engineering, University of Minnesota, Saint Paul, MN, United States; ^2^State Key Laboratory for Microbial Technology, Shandong University, Qingdao, China; ^3^Graduate School of Agricultural Sciences, Tohoku University, Miyagi, Japan

**Keywords:** fungi, gene regulation, plant biomass degradation, genetic mechanisms, fungal metabolism

## Introduction

Fungi in nature evolved distinctive capacities to deconstruct lignocellulose of plant biomass and dominate the carbon turnover process in terrestrial systems. Much of the net carbon sequestered by photosynthesis in land plants (5.6 × 10^13^ kg C/year) passes through fungi (Gilbertson and Ryvarden, 1987; Berbee et al., 2020). With plants as the reliable carbon and energy sources, lignocellulose-degrading fungi have evolved to survive in highly variable land environments and have become prevalent across the fungal kingdom, primarily in ascomycete and basidiomycete phyla (Kubicek et al., 2014; Rytioja et al., 2014). These fungi are seen as important for maintaining the sustainable ecosystem on our planet, and using them to convert plant biomass for producing advanced biofuels and bioproducts has been deemed as one of the most promising solutions to mitigate anthropogenic issues such as the climate change caused by the extensive use of fossil fuels (Kubicek, 2013).

Fungi use a complex lignocellulose-degrading system including several enzymes most of which are cataloged in the CAZy database (Carbohydrate-Active enZymes; http://www.cazy.org/) to degrade lignocellulose (Lombard et al., 2014). The composition of CAZyme repertories in different fungal species is usually shaped by the lifestyle that allows fungi to adapt to different environments and lignocellulose substrates. On the other hand, to deal with variable biotic (e.g., cooperative and competitive microbes) and abiotic (e.g., C and N source, pH, and light) factors, fungi have also evolved a sophisticated regulatory system to control the synthesis and secretion of these CAZymes precisely (Glass et al., 2013; Kubicek et al., 2014). Regarding this, probing the CAZyme encoding gene resources and dissecting the regulation of these genes are two main aspects of research toward understanding the genetic basis of lignocellulose-degrading fungi. The fast development of systems biology and genetic approaches has dramatically facilitated this research (Floudas et al., 2012; Miyauchi et al., 2020), and it is providing invaluable fundamental knowledge that is transformative for industrial biomass conversion and environmental sustainability.

## Aims and Objectives

In this topic, we aim to report the most recent progress related to genetic mechanisms of plant biomass degradation by fungi. This includes original research articles that use traditional genetics and functional genomics to study these fungal mechanisms. With this topic platform, we intend to discuss the current state of: (1) the discovery of crucial plant biomass-converting genetic resources by leveraging systems biology approaches and (2) the dissection of the pathways controlling the fungal degradation of plant biomass in pure cultures and during species' interactions.

## Significant Findings in This Topic

Discovery of genetic resources includes identifying the key pathways involved in fungal interactions during lignocellulose degradation. Interspecies interactions between fungal mycelia affect the degradation process of woody materials as it alters not only fungal community but also fungal physiological activities (Fukasawa et al., 2020). Previous studies have reported elevated CO_2_ emissions and oxidase activities in interacting mycelia, leading to a prediction that fungal interaction may activate the degradation of organic matter (Fukasawa et al., 2020). However, measuring the weight loss of wood substrates during fungal competition has been generating discrepancies, begging research for further exploring how fungal interactions would influence lignocellulose-degrading pathways (Fukasawa et al., 2020). In this topic, using multi-omics, Presley et al. explain why the increased enzyme activities are not causing wood degradation during mycelial interaction of two brown rot species. In their study, they found that the secreted enzymes in the interaction zone are dominated by enzymes for fungal cell wall digestion and secondary metabolite production, while plant cell wall-digesting enzymes are mainly found under non-interacting conditions. Given that the fungal decay types are a continuum rather than segregated, as indicated by another paper in this topic (Schilling et al.), it would be helpful to test alternative interactions between fungi with variable decay types to further look at these key lignocellulose-degrading pathways.

Given the extraordinary capacities in degrading recalcitrant wood structures, wood decay fungi were thought of as the natural microbial resource harboring efficient lignocellulose-degrading pathways (Gilbertson and Ryvarden, 1987; Floudas et al., 2012; Miyauchi et al., 2020). To elucidate these in more detail, Kölle et al. compared the genomic sequences of two strains of the brown rot fungus *Rhodonia placenta* with different wood decay rates, and they identified a set of genes and mutations that might contribute to the phenotypic differences. Moreover, using a co-expression gene network, Zhu et al. found a lytic polysaccharide monooxygenase that can cooperate with the oxidative reagents during brown rot.

Fungi from unique environments also harbor valuable lignocellulose-degrading enzymes. Here, Li et al. report 16 cellulolytic fungal species isolated from the unique geographic and climatic environments in the Qinghai-Tibet Plateau. Using comparative transcriptomics, they identified the key mechanism of cellulase production in one of the promising cellulase producers—*Trichoderma harzianum* LZ117. By studying the secretome of the thermophilic fungus *Chaetomium thermophilum*, Jiang et al. found a superior cellobiohydrolase I (CBHI) with higher activity and increased temperature stability compared to *T. reesei* CBHI. From Austrian soils, Hinterdobler et al. isolated 12 new *T. reesei* strains that are highly genetically variable and that produce higher levels of cellulase and xylanase. After SNP verification, the authors propose that their new isolates are unique to European temperate environments and that these would provide biotechnological potential for example for non-GMO strain improvement by species crossing.

The expression of genes encoding lignocellulose-degrading enzymes in filamentous fungi is controlled by complex gene regulatory networks involving transcription factors and protein kinases. These regulators also affect many other cellular metabolic processes, known as cross-pathway regulation (Glass et al., 2013; Kubicek et al., 2014; Rytioja et al., 2014). In this Research Topic, three regulators related to lignocellulose degradation were studied by genetic approaches. Among these, Zhang et al. identified a novel transcription factor in *Talaromyces pinophilus* that negatively regulates the expression of amylolytic and (hemi-)cellulolytic enzyme encoding genes. It was found that the deletion of this regulator also resulted in a reduction in conidiation. In the cellulase producing workhorse *T. reesei*, Beier et al. report the regulatory functions of a putative kinase, USK1, and found that it is required for normal production of both cellulases and a set of secondary metabolites. By a similar approach, but in *Podospora anserina*, Li et al. describe the pleiotropic functions of a conserved kinase, SNF1, in positively regulating the production of cellulases but negatively affecting sterigmatocystin synthesis. These findings highlight the importance of regulatory pathways in fungal degradation of lignocellulose, and also point into an interesting future direction to dissect the regulation balance between primary and secondary metabolism during lignocellulose degradation.

## Conclusion

The findings reported in this Research Topic are expanding our understanding of fungal mechanisms involved in lignocellulose degradation. We expect these to advance relevant ecological and genetic engineering research and to create effective solutions to current environmental and sustainability challenges.

## Author Contributions

All authors listed have made a substantial, direct, and intellectual contribution to the work and approved it for publication.

## Conflict of Interest

The authors declare that the research was conducted in the absence of any commercial or financial relationships that could be construed as a potential conflict of interest.

## Publisher's Note

All claims expressed in this article are solely those of the authors and do not necessarily represent those of their affiliated organizations, or those of the publisher, the editors and the reviewers. Any product that may be evaluated in this article, or claim that may be made by its manufacturer, is not guaranteed or endorsed by the publisher.
